# F-18 Labeled Vasoactive Intestinal Peptide Analogue in the PET Imaging of Colon Carcinoma in Nude Mice

**DOI:** 10.1155/2013/420480

**Published:** 2013-12-26

**Authors:** Dengfeng Cheng, Yuxia Liu, Hua Shen, Lifang Pang, Duanzhi Yin, Yongxian Wang, Shanqun Li, Hongcheng Shi

**Affiliations:** ^1^Department of Nuclear Medicine, Zhongshan Hospital, Fudan University, Shanghai 200032, China; ^2^Shanghai Institute of Medical Imaging, Shanghai 200032, China; ^3^Shanghai Institute of Applied Physics, Chinese Academy of Sciences, Shanghai 201800, China; ^4^Department of Respiratory Medicine, Zhongshan Hospital, Fudan University, Shanghai 200032, China

## Abstract

As large amount of vasoactive intestinal peptide (VIP) receptors are expressed in various tumors and VIP-related diseases, radiolabeled VIP provides a potential PET imaging agent for VIP receptor. However, structural modification of VIP is required before being radiolabeled and used for VIP receptor imaging due to its poor in vivo stability. As a VIP analogue, [R^8, 15, 21^, L^17^]-VIP exhibited improved stability and receptor specificity in preliminary studies. In this study, F-18 labeled [R^8,15,21^, L^17^]-VIP was produced with the radiochemical yield being as high as 33.6% ± 3% (decay-for-corrected, *n* = 5) achieved within 100 min, a specific activity of 255 GBq/**μ**mol, and a radiochemical purity as high as 99% as characterized by radioactive HPLC, TLC, and SDS-Page radioautography. A biodistribution study in normal mice also demonstrated fast elimination of F-18 labeled [R^8,15,21^, L^17^]-VIP in the blood, liver, and gastrointestinal tracts. A further micro-PET imaging study in C26 colon carcinoma bearing mice confirmed the high tumor specificity, with the tumor/muscle radioactivity uptake ratio being as high as 3.03 at 60 min following injection, and no apparent radioactivity concentration in the intestinal tracts. In addition, blocking experiment and Western Blot test further confirmed its potential in PET imaging of VIP receptor-positive tumor.

## 1. Introduction

As an advanced imaging technique, position emission tomography (PET) allows noninvasive detection and quantitative assay of the in vivo distribution profile of radioactive isotope-labeled probes and has been extensively used in the diagnosis and staging of various diseases [1, 2]. Both advanced imaging devices and imaging agents are essential for such a functional imaging approach. It is exactly the desirable nuclide properties of the electronic nuclide F-18 and the characteristics of polypeptide molecules, for example, susceptibility to modification, high target specificity, and the in vivo pharmacokinetics suitable for imaging, that have attracted much attention to PET imaging of F-18 labeled polypeptide molecules [3, 4]. Large number of preclinical and clinical PET imaging studies have been conducted for F-18 labeled polypeptides, suggesting promising clinical potential in both tumor and cardiovascular research areas [5–8].

As a neuroendocrine mediator, vasoactive intestinal peptide (VIP) provides various marked biological activities including vasodilation, respiratory stimulation, and increasing blood glucose level [9, 10]. In 1970, Said et al. for the first time separated and purified this polypeptide from swine small intestine and determined its amino acid composition [11, 12]. VIP exerts physiological functions mainly through its receptor, which can be generally classified as VIP1 receptor and VIP2 receptor based on the differences in receptor distribution and in the affinity between the receptor and its ligand [13]. Recent studies identified expression of high-density and high-affinity VIP receptors in the cellular membranes of the tumors including intestinal adenocarcinomas, carcinoids, small cell lung cancer, mammary duct carcinoma, insulinoma, papillary thyroid carcinoma, melanoma, neuroblastoma, chromophil tumor, and pituitary adenoma secreting adrenocorticotropic hormone (ACTH). The maximum number of binding sites (*B*_max⁡_) of the high-affinity VIP receptors in tumor tissues can be as high as 22.5–57.9-fold higher than in normal tissues [14, 15].

Since Virgolin initially reported use of ^123^I labeled VIP in the imaging study of tumor VIP receptors, several potential ^123^I, ^99 m^Tc, F-18, and ^64^Cu  labeled VIP analogues have been developed [16–25]. While these isotope-labeled VIP analogues demonstrated good tumor receptor specificity, a common problem is the poor in vivo stability of VIP polypeptide, which leads to low uptake by tumor tissues and a depressed signal-to-noise (S/N) ratio. Therefore, our early efforts mainly focused on the structural modification for improving in vivo stability of VIP polypeptide and a series of studies using F-18 labeled methodology [26]. We noted that, in comparison to native VIP, [R^8,15,21^, L^17^]-VIP (H-His-Ser-Asp-Ala-Val-Phe-Thr-Arg-Asn-Tyr-Thr-Arg-Leu-Arg-Arg-Gln-Leu-Ala-Val-Lys-Arg-Tyr-Leu-Asn-Ser-Ile-Leu-Asn-NH_2_) had higher receptor affinity and in vivo stability, and that, by optimizing the reaction conditions, [R^8,15,21^, L^17^]-VIP was successfully labeled by F-18 [27]. On the basis of the earlier work, the present study aimed to further confirm, incorporating micro-PET imaging and in vitro histological tests, the potential of F-18 labeled VIP analogues in the imaging of tumors expressing VIP receptors.

## 2. Materials and Methods

[R^8,15,21^, L^17^]-VIP peptide was synthesized by GL Biochem (Shanghai) Ltd., with the purity being greater than 90%; K_222_ (Kryptofix 222), anhydrous acetonitrile (water content < 50 ppm), and p-nitrobenzenesulfonyl chloride were purchased from Across Belgium; 4-fluorobenzoic acid, N-hydroxysuccinimide (NHS), N,N′-dicyclohexylcarbodiimide (DCC), ethyl-4-dimethylamine benzoate, CH_3_SO_3_CF_3_, O-(N-succinimide) N,N,N′,N′-tetramethyl urea tetrafluoroborate (TSTU), and N′N′-bis- acrylamide were purchased from Fluka Switzerland; tetrapropylammonium hydroxide (1 mol/L water solution) was purchased from Aldrich Germany; acetoacetate, dichloromethane, N-hexane, trifluoroacetic acid, acetonitrile, sodium hydroxide, and the reagents required for SDS-Page electrophoresis, and so forth, were all domestic analytical reagents (Shanghai Chemical Reagent Co., China); anti-VIP primary antibody (H-16) and horse radish peroxidase (HRP) labeled rabbit anti-goat secondary antibody were purchased from Santa Cruz Biotechnology, USA; DAB was purchased from Genetech (Shanghai, China); SDG-PAGE gel preparation kit, Western primary antibody diluent, second antibody diluent, Western WB, and Western blocking solution were purchased from Beyotime Institute of Biotechnology (Haimen, Jiangsu, China).

### 2.1. F-18 Labeling [R^8,15,21^, L^17^]-VIP

The labeled precursor N-succinimide-4-[F-18] fluorobenzoate (F-18-SFB) was synthesized in reference to our previous reports, with a radiochemical yield over 70% [26]. The resultant F-18-SFB was coupled with [R^8,15,21^, L^17^]-VIP through the process briefly described next: about 1850 MBq (50 mCi) F-18-SFB was dissolved in 50 *μ*L acetonitrile, to which was added 100 *μ*g [R^8,15,21^, L^17^]-VIP (in 200 *μ*L 0.1 M borax-boric acid buffer), and was allowed to react under room temperature for 20 min. After filtering, the reaction solution was separated through Dionex P680 summit HPLC (Dionex, USA) equipped with Bioscan flow-count detector (Bioscan, USA). LiChrosorb C18 column (10 *μ*m, 300 × 3.9 mm) was used as the chromatographic column. The HPLC system was eluted at 1 mL/min using the following gradient: 0–20 min, 25% acetonitrile (containing 0.1% trifluoroacetic acid), 75% water (containing 0.1% trifluoroacetic acid) to 70% acetonitrile (containing 0.1% trifluoroacetic acid), 30% water (containing 0.1% trifluoroacetic acid). The peak with *t*_*R*_ = 11.43 min was collected. The resultant solution was rotation-dried and redissolved in 0.9% NaCl injection. The target product named [F-18]FB-[R^8,15,21^, L^17^]-VIP was produced and characterized using Radio-TLC, Radio-HPLC, and SDS-Page electrophoresis incorporating radioautography.

### 2.2. Characterization of [F-18]FB-[R^8,15,21^, L^17^]-VIP by Radio-TLC, Radio-HPLC, and SDS-Page Electrophoresis Incorporating Radioautography

*Radio-TLC Method.* After sampling and spotting the silica plate, spread in methanol/water (V/V, 85/15), and the *R*_*f*_ value of the characterization product was obtained by scanning with Radio-TLC Scanner (Bioscan, USA). Radio-HPLC was performed as described under 2.1.

*Detailed Description of SDS-Page Electrophoresis*. The electrophoresis device included minivertical electrophoresis system, direct current stabilizing power, and gel imager, all produced by Bio-Rad. 10 *μ*L standard protein solution, 7.5 *μ*L [R^8,15,21^, L^17^]-VIP solution, and 7.5 *μ*L [F-18]FB-[R^8,15,21^, L^17^]-VIP solution (approximately 100,000 cpm) were separately loaded onto the adjacent wells on SDS gel. When electrophoresis had completed, the glass plate was pried open and the gel was placed on multipurpose energy storage phosphor screen for exposure for 30 min. The electrophoresis bands of the radioactive sample were obtained as scanned by Bio-Rad Cyclon system phosphor screen scanner. Finally, the gel was placed in large culture plates, to which staining solution was added, heated in micro oven for 30s, and allowed to stand for 2 min. The gel was rinsed several times with distilled water and destained with destaining solution until protein bands became distinct. The electrophoresis band of [R^8,15,21^, L^17^]-VIP was obtained by imaging in gel imaging system. The consistency between this band and the [F-18]FB-[R^8,15,21^, L^17^]-VIP band shown in radioautography was used for confirming identity of the product.

### 2.3. Normal Animal Distribution Test of [F-18]FB-[R^8,15,21^, L^17^]-VIP

All animal experiments followed the laboratory animal specifications of Zhongshan Hospital, Fudan University. In order to obtain preliminary in vivo pharmacokinetics data of this radiolabeled polypeptide, 100 *μ*L 3.7 MBq (100 *μ*Ci) [F-18]FB-[R^8,15,21^, L^17^]-VIP was injected into each normal BALB/C mouse through tail vein. 20 mice were assigned to 4 groups evenly. At 5, 30, 60, and 120 min following injection, they were sacrificed under anesthesia. Mice in each group were immediately subject to autopsy, where the organs and tissues of interest, including brain, liver, kidneys, heart, lungs, spleen, stomach, small intestine, muscles, bones, and blood, were collected. All the tissue samples were weighed and their counting were measured by *γ* counter, so that the percent injected dose per each gram of tissue (% ID/g) was derived.

### 2.4. [F-18]FB-[R^8,15,21^, L^17^]-VIP in Micro-PET Imaging Study in C26 Tumor Bearing Mice

C26 tumor bearing mice were obtained by right subaxillary implantation and were used in PET imaging test when the tumor had grown to the size of about 1 cm. PET imaging test in small animals was performed on a micro-PET R_4_ scanner (Concorde Microsystems, Siemens, Germany). After sodium pentobarbital anesthesia, C26 tumor bearing mice were injected with 1.5 MBq (40 *μ*Ci) [F-18]FB-[R^8,15,21^, L^17^]-VIP and placed in the central zone of the micro-PET device separately at 5 min, 60 min, and 120 min following injection. They underwent static scanning for 10 min at different points. The original data obtained were transformed into 2D image by Fourier transform and 2D FBP reconstruction.

Blocking experiment was conducted to explore whether [F-18]FB-[R^8,15,21^, L^17^]-VIP was receptor-specific. 100 *μ*g of nonlabeled VIP polypeptide mixed with 40 *μ*Ci [F-18]FB-[R^8,15,21^, L^17^]-VIP was injected into each C26 tumor bearing mouse. The imaging experiment with blocking was performed 60 min following injection, using scanning for 10 min and the same procedures and data treatment process as above.

### 2.5. Tumor Western Blot Test after Completion of Imaging

Upon completion of PET imaging, C26 colon carcinoma bearing mice were sacrificed. Tumor tissues were extracted and about 1 g was weighed. After chopping on ice, they were homogenized with 10 mL of Western Blot tissue lysis solution in a glass homogenizer. The homogenized mix solution was added into several 1 mL cylindrical tubes, which were centrifuged at 14000 g for 5 min. The supernatant was extracted and a small amount was subject to BCA protein assay. A final concentration of 10 *μ*g/*μ*L was produced to be used in Western Blot test, following the specific procedures in reference to literature [28].

## 3. Results

### 3.1. Isolation and Characterization of F-18 Labeled Polypeptide Product

The target product [F-18]FB-[R^8,15,21^, L^17^]-VIP produced by F-18 labeling achieved the radiochemical yield as high as 33.6% ± 3% (decay-for-corrected, *n* = 5) within 100 min, with a specific activity of 255 GBq/*μ*mol and a radiochemical purity as high as 99% as characterized by radioactive HPLC, TLC, and SDS-Page radioautography.

**Figure 1:**
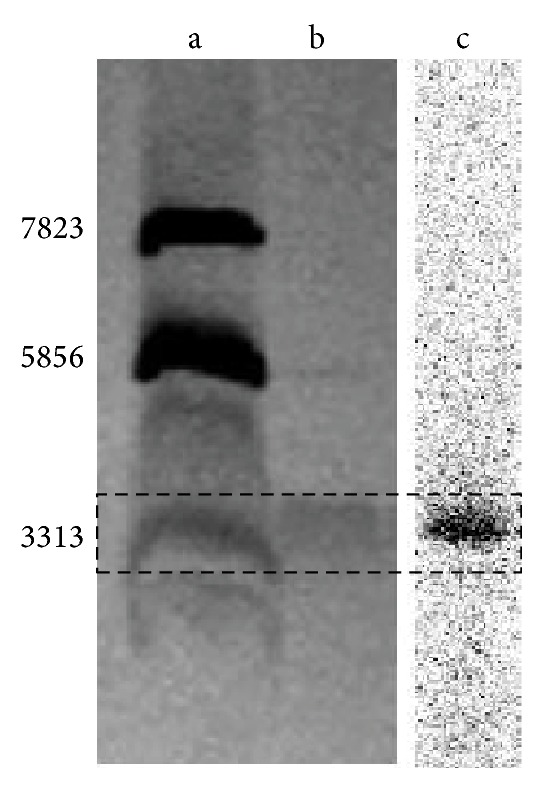
The electrophoresis band of the radioactive product (c) corresponded to the band of [R^8,15,21^, L^17^]-VIP (b) and the 3313 Dalton band of marker protein (a).

Through radioactive TLC, we noted that the *R*_*f*_ of both products was around 0.1, which was consistent with the TLC chromatographic characteristics of polypeptides, and no impurity peaks were identified. In the HPLC chromatograph, the retention time of [F-18]FB-[R^8,15,21^, L^17^]-VIP was 11.23 min, which was similar to that of the [R^8,15,21^, L^17^]-VIP UV absorption peak (about 11 min). As the stoichiometric amount of the radioactive product was too small (at approximately nanomole scale) to be characterized by ^1^H-NMR or MS, we further selected SDS-Page gel electrophoresis and radioautography for analysis. It was noted that the electrophoresis band of the radioactive product corresponded to the band of [R^8,15,21^, L^17^]-VIP and the 3313 Dalton band in marker (Figure 1), further suggesting that this was our target product. In this study, confirmation of purity and target product of F-18 labeled polypeptide by several characterization methods was reported for the first time, which will provide methodology guidance for the characterization of radiolabeled products of other polypeptides and antibodies.

### 3.2. Tissue Distribution Test in Normal Mice

**Figure 2:**
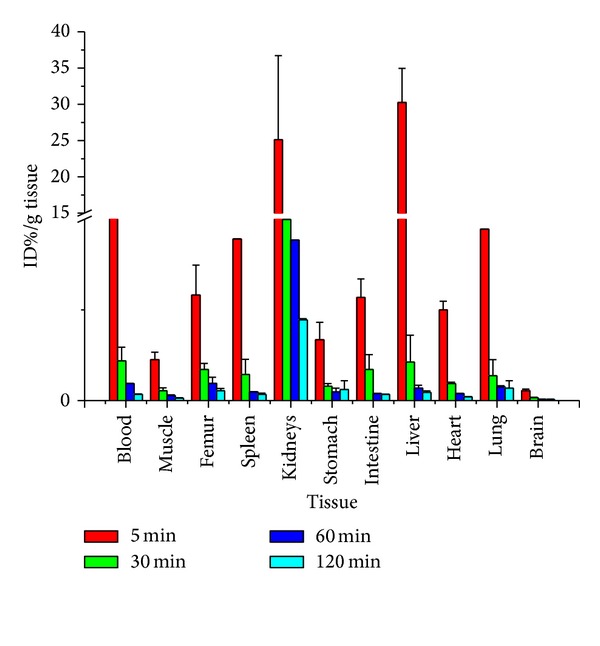
Biodistribution of [F-18]FB-[R^8,15,21^, L^17^]-VIP in normal Balb/C mice. Mice received an injection through the tail-vein of 100 *μ*Ci of radioligand. The animals were euthanized at 5, 30, 60, 120 min p.i. and biodistribution of radioligand was determined in the excised tissue. The results are presented as % injected dose per gram tissue (*Y* axle) and tissue (*X* axle). Data are presented as the mean ± SEM (*n* = 5 for each).

Results of the tissue distribution test (Figure 2) suggested low bone absorption, indicating good defluorination stability, which is a mandatory consideration in selecting F-18 PET imaging agent. In addition, data showed that, 5 min following injection, radioactivity had been quickly distributed in various organs, mostly in kidneys and liver tissues. This was primarily due to the fact that, after metabolizing in the blood and liver, polypeptide was quickly eliminated in the kidneys. At 30 min, the radioactivity concentration in the VIP expressing tissues, for example, lungs, liver, and intestines, was slightly higher than in other tissues but was by far lower than in the kidneys. By 120 min following injection, absorption by each organ had markedly reduced, indicating that the target product was consistent with the fast elimination characteristic of polypeptides in the organism.

### 3.3. Micro-PET Images and Results Analysis

**Figure 3:**
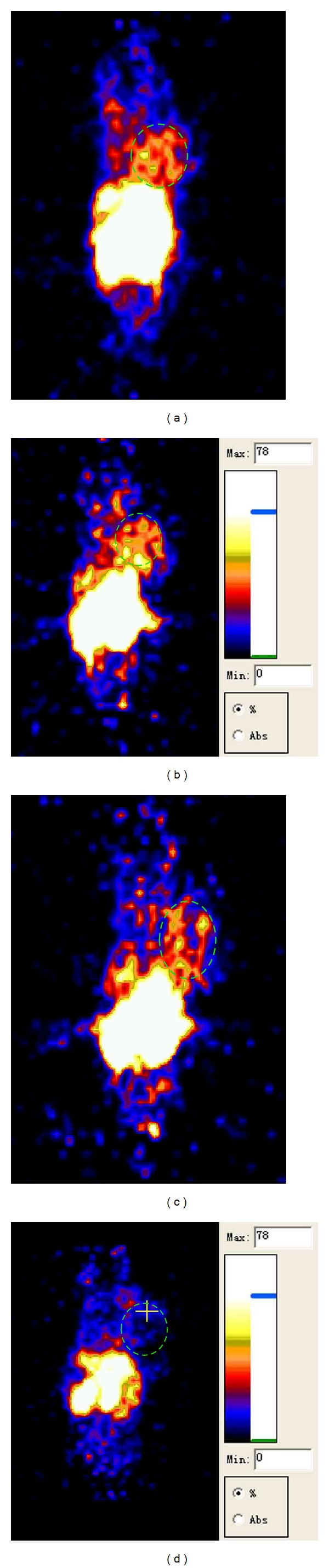
Micro-PET images of the same C26 tumored mouse obtained at 5 min (a), 60 min (b), and 120 min (c) after injection of 40 *μ*Ci [F-18]FB-[R^8,15,21^, L^17^]-VIP. Another mouse was injected with the mixture of 40 *μ*Ci [F-18]FB-[R^8,15,21^, L^17^]-VIP and 100 *μ*g VIP for blocking study; Figure 3(d) is showing the micro-PET image of this mouse at 60 min p.i. Circles indicated tumor area. All images are at the identical color threshold as being shown on the right.

Given the in vivo imaging advantage of PET imaging and based on our preliminary animal evaluation results, micro-PET imaging test for [F-18]FB-[R^8,15,21^, L^17^]-VIP in C26 tumor bearing mice was conducted and produced the imaging outcomes as shown in Figure 3. As can be seen from the images, the molecular probe maintained good in vivo defluorination stability, with no marked bone absorption observed up until 120 min. Furthermore, as it could not pass through the blood-brain barrier, very small amount entered the brain; absorption in the lungs was not marked, either. Radioactivity had been systemically distributed in the mice by 5 min and had achieved certain concentration at the tumor site, with the tumor-to-muscle ratio (T/M) being as high as 1.84. At 60 min following injection, with the exception of kidneys and liver, radioactivity had been eliminated from the majority of tissues. At that time the T/M could be as high as 3.03, and no marked radioactivity concentration was observed in the intestinal tracts. At 120 min following injection, the T/M had reached 3.74, the S/N ratio had further increased, and the radioactivity concentration in the liver had markedly reduced. In addition, it was noted in the blocking experiment that, at 60 min following injection, the radioactivity concentrations in the tumors of blocked mice had markedly reduced from those in the tumors of unblocked mice, with the T/M reduced to 1.01.

### 3.4. Western Blot Analysis of VIP Receptor Expression in the Tumor

**Figure 4:**
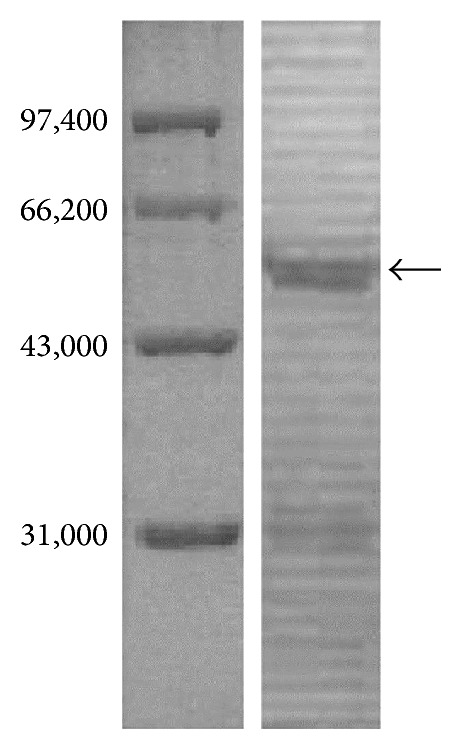
Western Blot analysis of the tumor tissue. The left electrophoresis bands are the marker protein and the right band is showing positive VIP receptor protein existing in the tumor tissue.

The purpose of this test was to further confirm that the micro-PET imaging outcome was specific radioactivity concentration, proving that the concentration signals were the result of [F-18]FB-[R^8,15,21^, L^17^]-VIP and the VIP receptors positively expressed in C26 colon carcinoma tissues. We noted from the test results that positively expressed VIP receptors presented in the tumor tissues (Figure 4).

## 4. Discussion

Like other neuropeptides, VIP is quickly metabolized in liver and excreted from kidneys. Given this, when VIP is to be used as an imaging agent, chemical modification of its structure is mandatory to extend the biological half-life in order to meet the imaging requirement. The method by Virgolini et al. and Hessenius et al. using I-123 labeling nonmodified VIP for the diagnosis of intestinal adenoma and endocrine tumor remains significantly controversial [16–19]. Pallela et al. labeled VIP analogue (TP3654) by ^99m^Tc and conducted animal distribution and imaging tests in nude mice transplanted with colon carcinoma cells LS174T [20]. It was observed that, despite the relatively depressed radioactivity concentration in tumor tissues with the absorption of 0.24 ± 0.08 (%ID/g) by tumor tissues at 4 h after injection, the retention time was relatively long, contributing to an absorption maintained at 0.23 ± 0.13 (%ID/g) at 24 h; in addition, radioactivity was quickly eliminated in blood and other nontarget organs, with the elimination phase half-life of 120 min. Therefore, at 4 h after injection, the tumor/muscle ratio (T/M) could be as high as 2.73 ± 1.09 and the tumor/blood ratio (T/B) could be as high as 1.16 ± 0.29; at 24 h, the T/M and T/B could be as high as 6.28 ± 3.09 and 1.98 ± 1.44, respectively. The results of the distribution test were compared with the test results on the same animal model: at 24 h after injection, in comparison to the T/M (0.38 ± 0.56) and T/B (0.88 ± 0.16) with ^125^I-VIP, ^99m^Tc-TP3654 resulted in substantial improvement.

While ^99m^Tc labeled VIP analogues have successfully undergone large number of preclinical human imaging studies, PET features ever-increasing application and the advantages in sensibility and definition, and so forth. PET imaging studies of VIP will be studied by different study teams. Tharkur et al. also synthesized a new VIP analogue TP3982, which was successfully labeled by ^64^Cu using (N_2_S-benzoyl)_2_ as the bifunctional chelating agent for animal distribution and micro-PET imaging studies in T47D breast tumor bearing mice [25]. The study confirmed that ^64^Cu-TP3982 not only maintained the biological activities of endogenous VIP, but also presented better protein degradation stability. Animal distribution study data showed that the absorption of ^64^Cu-TP3982 (17.04 ± 0.73 ID%/g) in tumor tissues was 74-fold higher than that of ^99m^Tc-TP3982 (0.23 ± 0.13 ID%/g), which was possibly the result of the very good in vivo stability of ^64^Cu-TP3982. In contrast, the chelate ^99m^Tc was likely to be oxidized into ^99m^Tcc^7+^ and thus quickly eliminated from the organism, leading to relatively low absorption in tumor and other tissues. In vivo PET imaging results showed very marked absorption of ^64^Cu-TP3982 in tumor tissues. ROI analysis showed that the intensity of radioactivity in tumor tissues at 4 h following injection was 9.15 ± 0.5 higher than in muscles and was even 13.9 ± 0.7-fold higher at 24 h, indicating a significantly superior imaging quality than with ^99m^Tc-TP3654 and ^99m^Tc-TP3982. A series of studies suggested ^64^Cu-TP3982 to be a promising PET imaging agent for tumors with high VIP receptor expression. However, while emitting *β* ^+^ (655 keV), ^64^Cu also emits *β* ^−^ (573 keV); the *β* ^+^ required for PET imaging only accounts for a small portion of 19% [1]. From the perspective of PET imaging, F-18 features desirable nuclide properties. Despite its short half-life relative to ^64^Cu, for a polypeptide with quick pharmacokinetics, the physical half-life of F-18 is more suitable for labeling polypeptide to meet the requirement of radiolabeling of polypeptide agents in PET [1]. However, in a PET imaging study of an F-18 labeled VIP analogue (Arg^15^, Arg^21^) VIP on T-47D nude mice model of human breast cancer, despite the T/M was as high as 3.4, the T/B was only 0.94. As can be seen from the study results, even with T/M being greater than 1, elevated blood background would also interfere with imaging quality, making it difficult to identify the lesion sites [24].

In summary, despite the limited reports of tumor VIP receptor imaging studies, a promising prospect of application has been preliminarily revealed: (1) VIP receptor imaging provides a highly sensitive and highly specific qualitative niveau diagnostic method for VIP receptor-positive tumors, gastrointestinal adenomas in particular, with better pharmacokinetics than in monoclonal antibody immune-imaging; (2) VIP receptor imaging is expected to be used for predicting the efficacy of VIP, VIP analogues, or VIP receptor antagonists on different tumors, helping the patients to select the treatment regimen; (3) VIP receptor imaging provides a noninvasive study approach for in-vitro revelation of the histological distribution and density of VIP receptors in the organism in physiological settings, which is vital for the study of the physiological, pathological, and pharmacological effects on certain tissues and organs.

## 5. Conclusions

In the present study, a novel VIP analogue was successfully labeled by F-18 and characterized by various methods, providing a detailed characterization methodology study of F-18 labeling polypeptides and antibodies. In addition, several key messages were obtained by micro-PET imaging. Firstly, the lung absorption significantly lower than tumor absorption level suggested a significant improvement from ^123^I-VIP, which was possibly the favorable result of improved stability. Secondly, the target specificity of [F-18]FB-[R^8,15,21^, L^17^]-VIP for colon carcinoma, a VIP receptor-positive tumor, was confirmed: high tumor absorption had been shown by 60 min, with the T/M up to 3.03. Over time, T/M had increased to 3.74 by 120 min. This further increase of T/M over time also predicts the target specificity of this molecular probe. In addition, the lowered liver background at 120 min was favorable for the imaging of liver metastasis of colon carcinoma. Furthermore, the blocking experiment at 60 min following injection and the Western Blot test of tumor tissues affirmed the specificity of [F-18]FB-[R^8,15,21^, L^17^]-VIP for VIP receptor-positive tumors from different perspectives, indicating a further improved imaging accuracy than in F-18-FDG PET imaging.
